# Age-related differences in symptoms, diagnosis and prognosis of bacteremia

**DOI:** 10.1186/1471-2334-13-346

**Published:** 2013-07-24

**Authors:** Astrid L Wester, Oona Dunlop, Kjetil K Melby, Ulf R Dahle, Torgeir Bruun Wyller

**Affiliations:** 1 Division of Infectious Disease Control, Norwegian Institute of Public Health, PO Box 4404, Nydalen, N-0403, Oslo Norway; 2 Medical Intensive Care Unit, Oslo University Hospital, Oslo, Norway; 3 Institute of Clinical Medicine, University of Oslo, Oslo, Norway; 4 Department of Microbiology, Oslo University Hospital, Oslo, Norway; 5 Department of Geriatric Medicine, Oslo University Hospital, Oslo, Norway

**Keywords:** Bacteremia, Sepsis, Elderly, Risk factor, Mortality, Organ failure

## Abstract

**Background:**

Elderly patients are at particular risk for bacteremia and sepsis. Atypical presentation may complicate the diagnosis. We studied patients with bacteremia, in order to assess possible age-related effects on the clinical presentation and course of severe infections.

**Methods:**

We reviewed the records of 680 patients hospitalized between 1994 and 2004. All patients were diagnosed with bacteremia, 450 caused by *Escherichia coli* and 230 by *Streptococcus pneumoniae*. Descriptive analyses were performed for three age groups (< 65 years, 65–84 years, ≥ 85 years). In multivariate analyses age was dichotomized (< 65, ≥ 65 years). Symptoms were categorized into atypical or typical. Prognostic sensitivity of CRP and SIRS in identifying early organ failure was studied at different cut-off values. Outcome variables were organ failure within one day after admission and in-hospital mortality.

**Results:**

The higher age-groups more often presented atypical symptoms (p <0.001), decline in general health (p=0.029), and higher in-hospital mortality (p<0.001). The prognostic sensitivity of CRP did not differ between age groups, but in those ≥ 85 years the prognostic sensitivity of two SIRS criteria was lower than that of three criteria. Classical symptoms were protective for early organ failure (OR 0.67, 95% CI 0.45-0.99), and risk factors included; age ≥ 65 years (OR 1.65, 95% CI 1.09-2.49), comorbid illnesses (OR 1.19, 95% CI 1.02-1.40 per diagnosis), decline in general health (OR 2.28, 95% CI 1.58-3.27), tachycardia (OR 1.50, 95% CI 1.02-2.20), tachypnea (OR 3.86, 95% CI 2.64-5.66), and leukopenia (OR 4.16, 95% CI 1.59-10.91). Fever was protective for in-hospital mortality (OR 0.46, 95% CI 0.24-0.89), and risk factors included; age ≥ 65 years (OR 15.02, 95% CI 3.68-61.29), ≥ 1 comorbid illness (OR 2.61, 95% CI 1.11-6.14), bacteremia caused by *S*. *pneumoniae* (OR 2.79, 95% CI 1.43-5.46), leukopenia (OR 4.62, 95% CI 1.88-11.37), and number of early failing organs (OR 3.06, 95% CI 2.20-4.27 per failing organ).

**Conclusions:**

Elderly patients with bacteremia more often present with atypical symptoms and reduced general health. The SIRS-criteria have poorer sensitivity for identifying organ failure in these patients. Advanced age, comorbidity, decline in general health, pneumococcal infection, and absence of classical symptoms are markers of a poor prognosis.

## Background

The incidence of sepsis in humans has been shown to increase with age [1-3]. Elderly patients are at particular risk for bacteremia and sepsis owing to multiple factors such as comorbid illnesses, immunosenescence, malnutrition, instrumentation and institutionalization [4]. Previous studies have identified age as an independent risk factor for death due to sepsis [3,5,6] and for severe bloodstream infections [7-12], although conflicting results are also reported [13].

The clinical presentation of sepsis is often atypical in elderly patients, complicating and potentially delaying diagnosis [4]. A decline in general health and unspecific functional deterioration, such as reduced ability to perform daily tasks, may be the only symptoms of severe illness, including sepsis [14]. Possible effects of age-related biological changes upon the clinical course or prognosis of sepsis are not well described. In addition, it is not known whether atypical presentation is predictive of severe sepsis or death when established criteria for sepsis and organ failure are used.

To address the special challenges regarding clinical evaluations of elderly patients with severe infection we studied 1) the clinical presentation and severity related to age, 2) age linked differences in prognostic sensitivity of C-reactive protein (CRP) and systemic inflammatory response syndrome (SIRS) for early organ failure, and 3) whether age and age-related clinical presentation are additional risk factors for early organ failure and death, in a mixed group of patients with community-acquired bacteremia caused by *E*. *coli* or *S*. *pneumoniae*.

## Methods

### Patients and setting

This study was conducted at Aker University Hospital in Oslo, Norway, between 1994 and 2004. During the study period, the hospital had 350 beds and served a population of 500,000 people for urology and abdominal vascular surgery, and 180,000 people for internal medicine, general surgery and psychiatry.

Medical records for all adult (≥ 16 years) patients admitted during the study period with culture-verified bacteremia due to *E*. *coli* or *S*. *pneumoniae* infection were retrieved from the hospital’s bacteriology laboratory database. Patients who had more than one episode of bacteremia during the study period were registered only once in the study. As we wanted to study community-acquired infections, we included only patients who had blood cultures drawn on the day of or day after hospital admission. Only patients with medical records available were included in the study.

### Clinical data

The following clinical data on comorbidities, risk factors for infection, diagnoses, signs and symptoms were extracted from medical records for all patients included in the study.

Comorbid illnesses specified in the medical records were extracted and categorized using a predefined list. Malignant disease was registered in cases of cancer or hematological malignancy. Alcoholism was registered when accompanied by organ involvement or social decompensation. Chronic renal failure was registered if repeated creatinine values > 500 μmol/L in preceding admissions, differentiated as severe if combined with dialysis or medication specific for renal failure, and as moderate chronic if neither dialysis nor medication specific for renal failure was recorded. Heart failure and cardiomyopathy were both registered as heart failure.

Risk factors for infection included having an indwelling urinary catheter, surgical procedure at site of infection within the two weeks prior to admission, obstruction of the gastrointestinal or urinary tracts, and chronic inflammation. Medication with implicit risk for infection included use of corticosteroids ≥ a dose equivalent to 10 mg prednisolone per day, chemotherapy in the two weeks before admission or other immunosuppressive medication on a daily basis.

Tentative diagnoses by the admitting physicians were categorized into infection, non-specific diagnoses (including delirium and acute deterioration in the ability to perform daily tasks), organ-specific diagnoses not indicating an infection (i.e. myocardial infarction, acute abdominal pain, acute asthma), and missing/others.

Symptoms indicative of infection preceding admission were dichotomized into “classical symptoms” and “atypical symptoms”. “Classical symptoms” included fever/chills, localized pain, nausea/vomiting, diarrhea, cough, dyspnea, expectoration, urinary urgency, painful voiding, hematuria, skin rash, coma, and seizures, whereas “atypical symptoms” included malaise, falls, dizziness, syncope, unsteadiness, immobility, acute urinary or fecal incontinence, paresis, speaking difficulties, and confusion.

Signs of infection in the emergency department (ED) included decline in general health if recorded. Findings during the physical examination indicative of localized pathology were recorded, and markers of systemic inflammatory response syndrome (SIRS) were registered according to international standards [15]. The SIRS criteria include body temperature more than 38.0°C or less than 36.0°C; heart rate more than 90 beats per minute; tachypnea manifested by a respiratory rate more than 20 breaths per minute or as a partial pressure of CO_2_ below 4.30 kPa; and a white blood cell count greater than 12,000/mm^3^ or below 4,000/mm^3^. The SIRS criteria were considered not met if data were not recorded. We used two alternative cut points for SIRS, ≥ 2 criteria met (SIRS-2) and ≥ 3 criteria met (SIRS-3). Cut points from the Simplified Acute Physiology Score (SAPS) [16] were used to define hypothermia (body temperature less than 36.0°C), fever (body temperature ≥ 38.5°C), leukocytosis (leukocyte counts above 15,000/mm^3^), and leukopenia (leukocyte counts below 3,000/mm^3^). C-reactive protein (CRP) values from blood samples drawn on the day of admission were categorized at 80 mg/L, which is applicable for predicting sepsis in patients with SIRS [17], and 200 mg/L, which is the suggested level for differentiating infection from other causes of shock [18]. We included new-onset atrial fibrillation as a marker of severe infection, as described previously [19].

Presumed primary site of infection was identified by one of the clinically trained authors (ALW) based on the medical history, symptoms, physical examination, blood tests, X-rays, specimen cultures from other body sites than blood, biopsies from surgical procedures, and autopsies. The sites of infection were categorized into urinary tract, lower respiratory tract, other (i.e. gastrointestinal tract, liver, pancreas and biliary tract, central nervous system), or inconclusive.

### Criteria for organ failure

Criteria for organ failure within one day after admission are presented in the Additional file 1. Whenever possible, criteria were defined according to the Sequential Organ Failure Assessment (SOFA) score system (cut point 2 or 3) [20]. Indicators for organ dysfunction, defined in the diagnostic criteria for sepsis in 2001 [21] and for severe sepsis and septic shock in 1992 [15], were also used. Criteria for acute renal failure were adjusted to the modified risk, injury, failure, loss and end-stage kidney (RIFLE) criteria [22], and on clinical presentation. Since the central nervous system is included in organ failure scoring systems for use in sepsis [23], we included impaired consciousness as an indicator of organ failure. However, signs of delirium were not included, because data on this state were not routinely collected upon admission. Data on liver and hematological markers as well as markers of peripheral perfusion such as serum lactate were not systematically registered in patient records, and were therefore excluded.

### Date of death

Date of death during hospitalization was extracted from patient records. For analytical purposes, mortality was classified into early hospital mortality (within ≤ 3 days of admission), and in-hospital death within 14 days of admission. Prior to the data extraction process survival after discharge from hospital had been confirmed through the National Population Register by the medical record staff. If death had occurred after the index stay, they had put the date onto the records.

### Statistical methods

In order to study any systematic differences in clinical presentation related to the oldest patients, descriptive analyses were performed for three age groups (< 65 years, 65–84 years and ≥ 85 years). In the multivariate analyses, however, age was dichotomized (< 65 and ≥ 65) based on preliminary analyses. Categorical variables were presented as absolute numbers and percentages and compared using Chi-squared tests. Normally distributed numerical variables were compared using one-way ANOVA, and non-normal variables using Kruskal-Wallis tests and Mann–Whitney tests. The number of “classical” symptoms was dichotomized at three symptoms, “atypical” symptoms were dichotomized at one symptom.

Non-parametric correlation analysis (Spearman rho) was performed to study the relationship between CRP value at admission and the number of failing organs within one day of admission. The associations between organ failure and different cut-points of CRP and different number of SIRS criteria were explored using Chi-squared tests.

In order to identify factors recorded upon admission to the ED independently associated with either early organ failure or in-hospital death (truncated at 14 days after admission to hospital), variables significantly associated with these outcomes (p < 0.05) in bivariate analyses were entered into binary logistic regression models. Ordinal factors not linearly associated with either of the two outcomes were dichotomized. For variable selection, we used backward stepwise removal of variables based on likelihood-ratio judgments. Model summary given in Nagelkerke R square and model of fit given by the Hosmer and Lemeshow test were applied. We also tested for any interactions between the dichotomized age variable and the other factors in the full main effects models. To obtain the logit of the two outcomes when interactions were active, the macro Modprobe developed and adjusted to SPSS by Hayes and Matthes was applied [24]. However, since the statistical power of interaction analyses is generally low, the effects of interacting variables on outcomes are presented only as directions rather than graphically or by numbers.

One-year survival by number of early failing organs, bacterial species, and age were analyzed using Kaplan Meier survival analysis, applying the log-rank test. A Kaplan Meier plot was used to present the results graphically. All analyses were performed with SPSS 17.0 software (SPSS, Chicago, IL).

### Ethical considerations

The study was approved by the South-East Norway Regional Committee for Ethics in Medical Research. The Norwegian Data Inspectorate gave permission to carry out the study without the patient consent. Dispensation of professional confidentiality was given by the Norwegian Directorate of Health.

## Results

Between 1994 and 2004, 1150 patients had a blood culture positive for either *E*. *coli* or *S*. *pneumoniae*. Of these, 759 had the positive blood culture drawn on the day of admission or the following day. For 79 patients the clinical data was either unavailable or inadequate for analyses. In total, a cohort of 680 patients was eligible for the study.

Table 1 presents basic characteristics, comorbid illnesses and clinical presentation by age group. The two oldest age groups had more comorbid illnesses and were more often admitted with non-specific tentative diagnoses than the youngest group. The two oldest age groups also differed from the youngest group by less frequently having “classical” symptoms and more frequently having “atypical” symptoms. In addition, the two oldest age groups presented more often with decline in general health, new-onset atrial fibrillation and reduced consciousness than the youngest group. Table 2 describes severity of infection by age group. The mean number of failing organs within one day after admission was significantly higher in the middle group than in the youngest age group. For the two oldest age groups, the site of infection was more difficult to determine than the youngest group. Furthermore, the two oldest age groups died earlier after admission and had higher in-hospital and one-year mortality than the youngest group.

**Table 1:** Descriptive data and clinical presentation by 3 age groups

		**Age groups (% within age group)**			
	**Total material (680 patients)**	**< 65 years** **(228 patients)**	**65**-**84 years** **(334 patients)**	**≥ 85 years** **(118 patients)**	**Overall p-value**
Age in years; median (IQR)	75 (57.5 - 82)	50.5 (38–58)	78 (73–81)	88 (86–91)	
Gender; male	289 (42.5%)	93 (40.8%)	160 (47.9%)	36 (30.5%)	0.004
Bacteraemia caused by E. coli	450 (66.2%)	131 (57.5%)	240 (71.9%)	79 (66.9%)	0.002
Bacteremia caused by S. pneumoniae	230 (33.8%)	97 (42.5%)	94 (28.1%)*	39 (33.1%)	
Comorbidity					
Number of comorbid conditions^1^; median (IQR)	1 (0–2)	0 (0–1)	1 (0–2)*	1 (1–2)*	< 0.001
Aftrial fibrrillation (chronic or paroxystic)	75 (11.2%)	0	47 (14.2)*	28 (24.3)*	< 0.001
Ischemic heart disease	145 (21.6%)	13 (5.8%)	91 (27.5%)*	41 (35.7%)*	< 0.001
Congestive heart failure	91 (13.5%)	6 (2.7%)	55 (16.6%)*	30 (26.1%)*	< 0.001
Hypertension	151 (22.5%)	25 (11.1%)	91 (27.5%)*	35 (30.4%)*	< 0.001
Cerebrovascular disorder	93 (13.8%)	11 (4.9%)	59 (17.8%)*	23 (20.0%)*	< 0.001
Chronic obstructive lung disease	71 (10.6%)	15 (6.6%)	50 (15.1%)*	6 (5.2%)	0.001
Alcohol abuse	46 (6.8%)	24 (10.6%)	21 (6.3%)	1 (0.9%)*	0.003
Malignant disease (solid cancer, leukemia or lymphoma)	45 (6.7%)	16 (7.1%)	24 (7.3%)	5 (4.3%)	0.540
Chronic renal failure	21 (3.1%)	6 (2.7%)	12 (3.6%)	3 (2.6%)	0.763
Diabetes mellitus	82 (12.2%)	20 (8.8%)	49 (14.8%)*	13 (11.3%)	0.103
Tentative diagnosis by admitting physician:					
Infection	331 (48.7%)	132 (57.9%)	143 (42.8%)*	56 (47.5%)	0.002
Non-specific^2^	89 (13.1%)	7 (3.1%)	59 (17.7%)*	23 (19.5%)*	< 0.001
Organ focused	158 (23.2)	56 (24.6%)	78 (23.4%)	24 (20.3%)	0.676
Symptoms on infection					
Total number of reported symptoms; median (IQR)	3 (2–4)	3 (2–4)	3 (2–4)	3 (2–3)	0.351
“Classical symptoms”^3^ ≥ 3	253 (37.4%)	110 (48.7%)	108 (32.5%)*	35 (29.7%)*	< 0.001
Zero ”classical symptoms”	57 (8.4%)	7 (3.1%)	36 (10.8%)*	14 (11.9%)*	0.002
“Atypical symptoms”^4^ ≥ 1	337 (49.6%)	82 (36.0%)	180 (53.9%)*	75 (63.6%)*	< 0.001
Signs of infection at admission					
Decline in general health	289 (42.6%)	81 (35.5%)	152 (45.6%)*	56 (47.5%)*	0.029
Leukocytosis (≥ 15000/μl)	280 (41.2%)	98 (43.0%)	129 (38.6%)	53 (45.3%)	0.363
Leukopenia (< 3000/μl)	37 (5.4%)	8 (3.5%)	24 (7.2%)	5 (4.3%)	0.140
C-reactive protein (CRP)					
CRP median (IQR); mg/L	191 (83–311)	222 (92 -351)	179 (72-266)*	180 (94-317)	0.022
CRP ≥ 80 mg/L	514 (75.6%)	175 (76.8%)	245 (73.4%)	94 (79.7%)	0.344
CRP ≥ 200 mg/L	324 (47.6%)	123 (53.9%)	147 (44.0%)	54 (45.8%)	0.062
Fever (≥ 38.5°C)	438 (65.8%)	154 (69.1%)	208 (64.0%)	76 (64.4%)	0.445
Hypothermia (< 36°C)	10 (1.5%)	7 (3.1%)	1 (0.3%)*	2 (1.7%)	0.027
Median number of SIRS criteria (IQR)	3 (2–3)	3 (2–3)	2 (2–3)	2 (2–3)	0.099
No. of patients with SIRS ≥ 3	343 (50.4%)	127 (55.7%)	160 (47.9%)	56 (47.5%)	0.149
No. of patients with SIRS ≥ 2	563 (82.8%)	198 (86.8%)	270 (80.8%)	95 (80.5%)	0.139
New-onset atrial fibrillation	104 (15.5%)	11 (4.9%)	72 (21.8%)*	21 (18.3%)*	< 0.001

^1^ Comorbid conditions: Malignant disease, alcoholism, diabetes mellitus, chronic renal failure, heart failure. ^2^ Non-specific tentative diagnoses: acute deterioration of performing daily tasks, reduced general condition, confusion, dizziness, falls, fainting, question of cerebral infarction. ^3^ “Typical symptoms”: fever/chills, localized pain, nausea/vomiting, diarrhoea, cough, dyspnoea, expectoration, signs of urinary tract infection (UTI) such as urgency, pyuria, haematuria; skin rash, coma, seizures. ^4^ “Atypical symptoms”: Malaise, fall/dizziness/syncope/unsteadiness, immobility, acute incontinence of urine or faeces, paresis, speaking difficulties, confusion. *Significantly different from the age group < 65, which was treated as reference group.

**Table 2:** Site and severity of infection by 3 age groups

		**Age groups (% within age group)**			
	**Total material (680 patients)**	**< 65 years** **(228 patients)**	**65**-**84 years** **(334 patients)**	**≥ 85 years** **(118 patients)**	**Overall p-value**
Acute organ failure within 1 day after admission					
No. of acute organ failures; mean (95% CI))	0.69 (0.62 – 0.76)	0.53 (0.41 – 0.64)	0.80 (0.70 – 0.90)*	0.70 (0.53-0.88)	0.003
Presence of ≥ 1 organ failure	290 (45.9%)	75 (35.2%)	165 (53.1%)*	50 (46.4%)	< 0.001
Type of acute organ failure					
Cardiovascular failure	80 (11.8%)	21 (9.3%)	50 (15.0%)*	9 (7.6%)	0.035
Respiratory failure	192 (28.2%)	54 (23.7%)	102 (30.5%)	36 (30.5%)	0.173
Acute renal failure	30 (4.5%)	6 (2.7%)	20 (6.1%)	4 (3.4%)	0.144
Coagulation failure	54 (8.4%)	14 (6.5%)	32 (10.1%)	8 (7.3%)	0.299
(platelets < 100x10^3^/mm^3^)					
Reduced consciousness	99 (14.6%)	19 (8.3%)	55 (16.5%)*	25 (21.4%)*	0.002
Sepsis-diagnosis at discharge					
Sepsis as main or side diagnosis (ICD-9 or ICD-10)	313 (46.0%)	86 (37.7%)	171 (51.2%)*	56 (47.5%)	0.007
Site of infection					
Urinary tract	255 (37.5%)	84 (36.8%)	132 (39.5%)	39 (33.1%)	0.445
Respiratory system	228 (33.5%)	94 (41.2%)	95 (28.4%)*	39 (33.1%)	0.007
Other sites^1^	107 (15.7%)	32 (14.0%)	61 (18.3%)	14 (11.9%)	0.179
Inconclusive	90 (13.2)	18 (7.9%)	46 (13.8%)*	26 (22.0%)*	0.001
Hospital stay					
Department responsible at admission:					
Surgical department (urology included)	138 (20.3%)	60 (26.3%)	61 (18.3%)	17 (14.4%)	0.006
Medical department	525 (77.2%)	161 (70.6%)	268 (80.2%)	96 (81.4%)	
Other departments; and missing	17 (2.5%)	7 (3.1%)	5 (1.5%)	5 (4.2%)	
Transferred to an ICU within 1 day after admission	153 (22.5%)	55 (24.1%)	82 (24.6%)	16 (13.6%)*	0.038
Length of stay in days; median (IQR)	8 (5–14)	7 (5–11)	9 (6–16)*	9 (5–15)	0.008
In-hospital mortality					
Total in-hospital mortality	92 (13.5%)	12 (5.3%)	62 (18.6%)*	18 (15.3%)*	<0.001
Day of in-hospital death; median (IQR)	5 (1–12)	16.5 (2.25 - 22.75)	6 (1–11.25)	2 (0.75 - 7)	0.065
Early in-hospital mortality (within 3 days after admission)	39 (5.7%)	4 (1.8%)	23 (6.9%)*	12 (10.2%)*	0.003
In-hospital mortality within 14 days after admission	72 (10.6%)	6 (2.6%)	51 (15.3%)*	15 (12.7%)*	<0.001
One-year mortality	168 (24.7%)	30 (13.2%)	101 (30.3%)*	37 (31.4%)*	<0.001

^1^ Gastrointestinal tract, liver, pancreas and biliary tract. * Significantly different from the age group < 65, which was treated as reference group.

**Figure 1:**
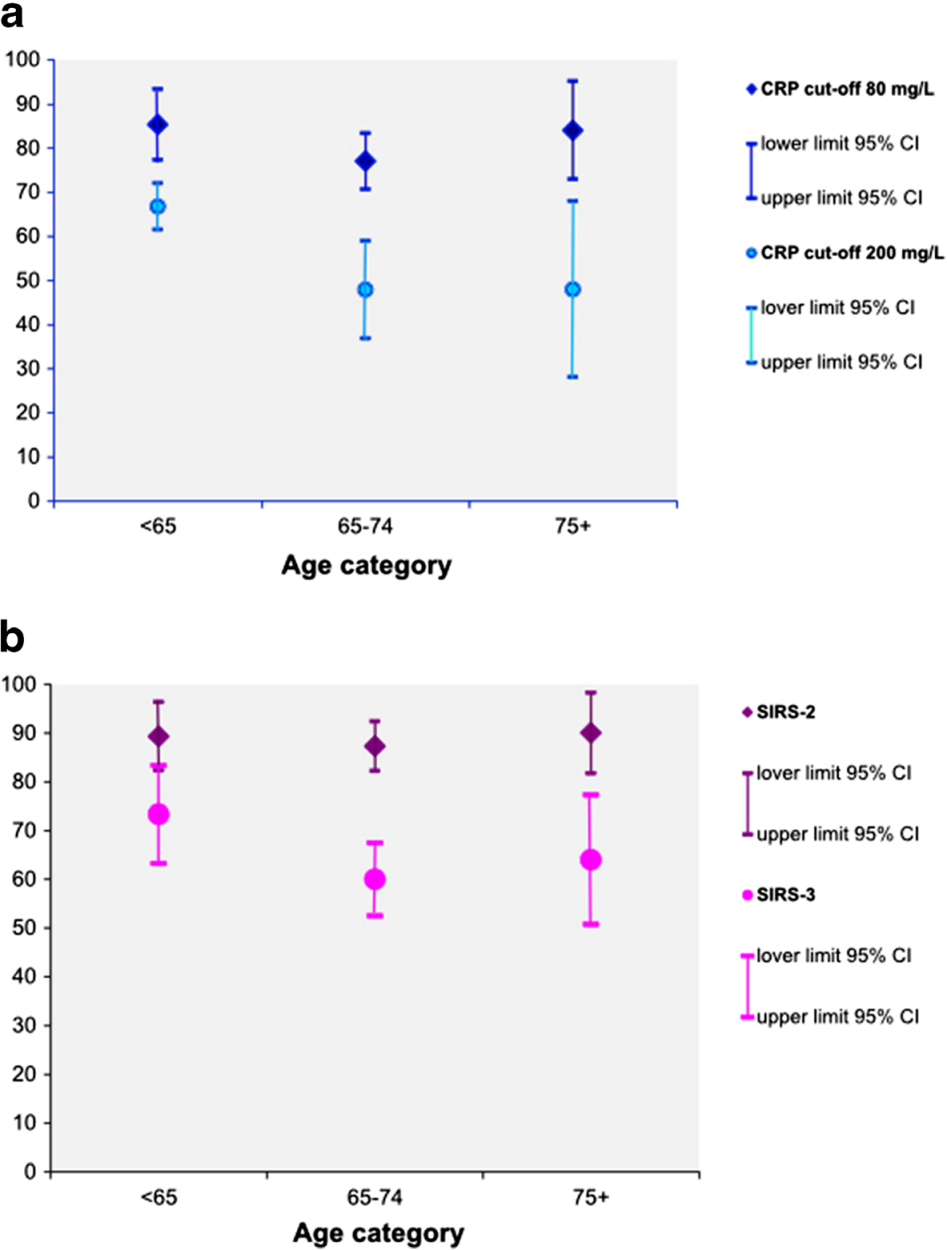
**Sensitivity of inflammatory markers for identifying ≥ 1 organ failure in bacteremia.** Prognostic sensitivity of **a)** C-reactive protein (CRP) and **b)** Systemic Inflammatory Response Syndrome (SIRS). CI = confidence interval.

The CRP values at admission were significantly correlated to the number of failing organs within one day after admission (r_s_ = 0.13, p = 0.001). Figure 1 shows the prognostic sensitivity with 95% confidence intervals of initial CRP value and of SIRS at different cut-off values in predicting ≥1 organ failure by age group. The prognostic sensitivity of a CRP value above 200 mg/L was lower in the middle age group than in the youngest group, whereas no age-associated differences were seen at cut-off value 80 mg/L. The prognostic sensitivity of SIRS-2 was lower than that of SIRS-3 for the two oldest age groups, but not for the youngest age group.

In Table 3, predictors for early organ failure available at admission are presented. Sufficient data on organ failure were available for 632 patients. As can be seen, age over 65 years, number of comorbid illnesses, more than three “classical” symptoms present, decline in general health, tachypnea and/or hyperventilation, and leukopenia remained as independent and statistically significant predictors in the multivariate model. The model contributed moderately to the prediction of having one or more failing organs (Nagelkerke R^2^ = 0.289), and fitted the data well (χ^2^ = 9.42, p = 0.30). Advanced age significantly reduced the effect of tachypnea and/or hyperventilation and the number of comorbid illnesses on the risk for early organ failure.

**Table 3:** **Information available at admission predictive for** ≥ **1 organ failure within one day**

	**Patients without organ failure(342 patients;% within this group)**	**Patients with organ failure(290 patients;% within this group)**	**Bivariate analyses OR(95% CI)**	**Multivariate analysis**; full main effects’model*OR(95% CI)
Age ≥ 65 years	204 (59.6)	215 (74.1)	1.94 (1.38-2.73)	1.65 (1.09 - 2.49)
Male gender	137 (40.1)	135 (46.6)	1.30 (0.95 – 1.79)	
No. of comorbid illnesses	-	-	1.41 (1.23-1.61)	1.19 (1.02 - 1.40)
Corticosteroid on a daily basis^1^	8 (2.4)	23 (8.0)	3.55 (1.56 – 8.05)	2.75 (1.10 - 6.89)
Cytostatic treatment within last 14 days	1 (0.3)	6 (2.1)	7.10 (0.85 - 59.34)	
Immunosuppressive treatment	1 (0.3)	5 (1.7)	5.90 (0.69 - 50.77)	
Number of symptoms ≥ 5	35 (10.2)	41 (14.2)	1.45 (0.90 – 2.35)	
“Classical symptoms”^2^ ≥ 3	141 (41.3)	97 (33.7)	0.72 (0.52 - 0.99)	0.67 (0.45 - 0.99)
“Atypical symptoms”^3^ ≥ 1	151 (44.2)	169 (58.3)	1.77 (1.29 - 2.42)	
Decline in general health (as described in the emergency department)	112 (32.8)	166 (57.2)	2.74 (1.98 - 3.79)	2.28 (1.58 - 3.27)
Heart rate > 90 beats per minute	197 (58.5)	203 (70.7)	1.72 (1.23 - 2.40)	1.50 (1.02 - 2.20)
Fever (≥ 38.5°C)	229 (68.4)	178 (62.2)	0.76 (0.55 - 1.06)	
Hypothermia (< 36°C)	4 (1.2)	6 (2.1)	1.77 (0.50 - 6.35)	
Tachypnoe and/or hyperventilation	78 (22.8)	167 (57.6)	4.60 (3.26 - 6.48)	3.86 (2.64 - 5.66)
Leukocytosis (≥ 15000/μl)	145 (42.4)	118 (40.7)	0.93 (0.68 - 1.28)	
Leukopenia (< 3000/μl)	6 (1.8)	31 (10.7)	6.70 (2.76 - 16.31)	4.16 (1.59 - 10.91)
CRP ≥ 80 mg/L	253 (74.0)	233 (80.3)	1.44 (0.99 - 2.10)	
CRP ≥ 200 mg/L	157 (45.9)	153 (52.8)	1.32 (0.96 -1.80)	
New-onset atrial fibrillation	35 (10.4)	64 (22.1)	2.45 (1.57 - 3.82)	

OR = odds ratio, CI = confidence interval. ^1^Equivalent to prednisolone ≥ 10 mg per day, ^2^“Classical symptoms”: fever/chills, localized pain, nausea/vomiting, diarrhea, cough, dyspnea, expectoration, urgency, painful voiding, hematuria, skin rash and coma/seizures, ^3^“atypical symptoms”: Malaise, falls, dizziness, syncope, unsteadiness, immobility, acute incontinence of urine or feces, paresis/speaking difficulties, acute confusional state. *Analyses of interactions indicated that high age significantly reduced the effect of tachypnea and/or hyperventilation and the effect of number of comorbid illnesses on early organ failure (results not shown).

Table 4 shows risk factors for in-hospital death within 14 days after admission. Age over 65 years, comorbidity, bacteraemia with pneumococci (rather than *E*. *coli*), leukopenia and number of failing organs within one day after admission all remained as independent risk factors for death, whereas having fever was protective. The model contributed moderately to the prediction of hospital mortality (Nagelkerke R^2^ = 0.428), and fitted the data well (χ^2^ = 3.2, p = 0.92). Advanced age significantly increased the effect of type of bacterium on hospital mortality. Type of tentative diagnoses before admission was not associated with mortality, whereas having “atypical symptoms” was significant only in bivariate analysis.

**Table 4:** **Predictive factors for in**-**hospital death within 14 days after admission**

	**Alive at 14 days(608 patients;% within this group)**	**Patients that died within 14 days(72 patients;% within this group)**	**Bivariate analyses OR(95% CI)**	**Multivariate analysis**; full main effects model*OR(95%CI)
Age ≥ 65 years	386 (63.5)	66 (91.7)	6.33 (2.70 - 14.83)	15.02 (3.68 - 61.29)
Male gender	246 (40.5)	43 (59.7)	2.18 (1.33 - 3.59)	1.93 (1.002 - 3.70)
S. pneumoniae	196 (32.2)	34 (47.2)	1.88 (1.15 - 3.08)	2.79 (1.43 - 5.46)
Comorbid illnesses ≥ 1	355 (58.1)	59 (81.9)	3.27 (1.76 – 6.09)	2.61 (1.11 - 6.14)
Immunmodulating and other medication				
Warfarin	51 (8.5)	5 (7.0)	0.82 (0.32 - 2.12)	
Corticosteroids^1^ on a daily basis	28 (4.7)	6 (8.5)	1.89 (0.75 - 4.73)	
Cytostatic treatment within last 14 days	5 (0.8)	2 (2.8)	3.46 (0.66 - 18.15)	
Immunosuppressive drugs	6 (1.0)	1 (1.4)	1.42 (0.17 - 11.94)	
Number of symptoms				
Number of symptoms ≥ 5	69 (11.4)	8 (11.3)	0.99 (0.45 – 2.15)	
“Classical symptoms”^2^ ≥ 3	236 (39.0)	17 (23.9)	0.49 (0.28 – 0.87)	
“Atypical symptoms”^3^ ≥ 1	296 (48.4)	44 (61.1)	1.67 (1.02 – 2.76)	
Prehospital tentative diagnosis				
Infection	303 (49.6)	28 (38.9)	0.65 (0.39 – 1.07)	
Non-specific^a^	81 (13.3)	8 (11.1)	0.82 (0.38 – 1.77)	
Site of infection				
Urinary tractus	241 (39.6)	14 (19.4)	0.37 (0.20 - 0.67)	
Lower respiratory tractus	193 (31.7)	35 (48.6)	2.03 (1.24 - 3.33)	
Others^b^	95 (15.6%)	12 (16.7%)	1.08 (0.56 – 2.08)	
Inconclusive	79 (13.0)	11 (15.3)	1.21 (0.61 - 2.39)	
Local immunocompromizing condition (at site of infection)				
Significant anatomical or physiological abnormality	206 (38.9)	17 (27.9)	0.61 (0.34 - 1.09)	
Surgical procedure performed within 14 days prior to admittance	54 (8.9)	1 (1.4)	0.15 (0.02 - 1.11)	
Signs of infection in the emergency department				
Fever (≥ 38.5°C)	402 (67.2)	36 (52.9)	0.55 (0.33 - 0.91)	0.46 (0.24 - 0.89)
Hypothermia (< 36 ° C)	7 (1.2)	3 (4.2)	3.90 (0.98 – 15.44)	
Tachycardia	383 (63.6)	44 (63.8)	1.01 (0.60 – 1.69)	
Tachypnoe and/or hyperventilation	215 (35.3)	40 (55.6)	2.29 (1.40 - 3.75)	
Leukocytosis (≥ 15000/μl)	255 (41.9)	25 (35.2)	0.75 (0.45 – 1.26)	
Leukopenia (< 3000/μl)	21 (3.5)	16 (22.5)	8.13 (4.01 - 16.49)	4.62 (1.88 - 11.37)
CRP ≥ 80 mg/L	457 (75.2)	57 (79.2)	1.26 (0.69 – 2.28)	
CRP ≥ 200 mg/L	280 (46.1)	44 (61.1)	1.84 (1.12 – 3.04)	
Decline in general health	244 (40.2)	45 (62.5)	2.48 (1.50 - 4.10)	
New-onset atrial fibrillation	84 (14.0)	20 (28.2)	2.41 (1.37 - 4.25)	
Number of failing organs within one day after admission	-	-	3.25 (2.48 - 4.26)	3.06 (2.2 - 4.27)

OR = odds ratio, CI = confidence interval. ^1^Equivalent to prednisolone ≥ 10 mg per day ^2^“Classical symptoms”: fever/chills, localized pain, nausea/vomiting, diarrhea, cough, dyspnea, expectoration, urgency, painful voiding, hematuria; skin rash and coma/seizures, ^3^“Atypical symptoms”: Malaise, falls, dizziness, syncope, unsteadiness, immobility, acute urinary or fecal incontinence, paresis/speaking difficulties, acute confusional state ^a^including delirium and acute deterioration in the ability to perform daily tasks ^b^gastrointestinal tract, liver, pancreas and biliary tract.

*Interaction analyses indicated that high age significantly increased the effect of type of bacterium on hospital mortality (results not shown).

**Figure 2:**
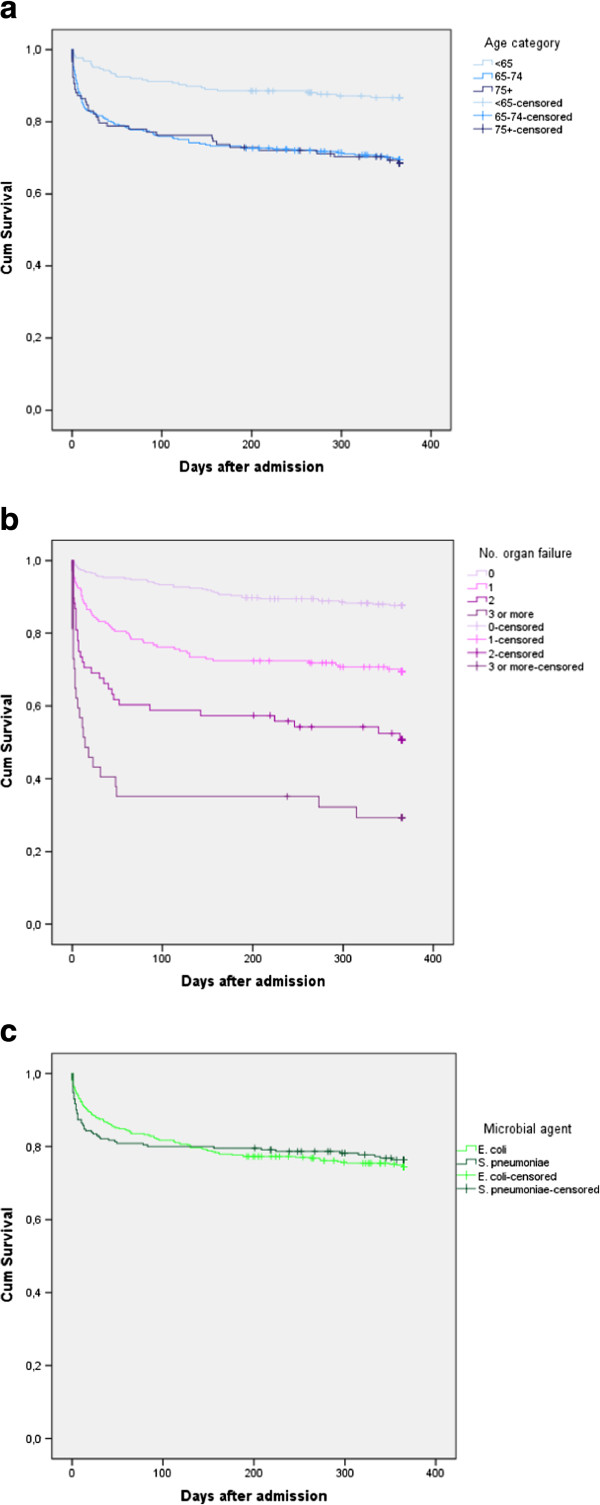
**Survival plots.** Kaplan-Meier survival estimates for one-year survival in days, by **a)** age group, **b)** number of organ failures; 3 = failure of three or more organs, and **c)** microbial agent.

Figure 2 displays Kaplan Meier plots of one-year survival curves by age group, number of failing organs and microbial agent. There were significant differences in one-year survival for age and number of failing organs (p < 0.001 for both), but not for type of bacteria (p = 0.75).

## Discussion

In this material, comprising nearly 700 patients with bacteraemia caused by *E*. *coli* or *S*. *pne*umoniae, several results indicate that age affects the clinical presentation, diagnostic markers, and outcome of severe infection.

Elderly patients more often presented with “atypical” symptoms like confusion, falls, malaise, incontinence and immobility, whereas “classical” symptoms of infection were more common among younger patients. The ED doctor’s impression of decline in general health was also a more frequent sign among the older patients. This reflects the general perception in geriatric care [25], but has, to our knowledge, not previously been confirmed in a large cohort of bacteremic patients. Older patients die earlier during hospitalization than younger patients [3], and are more rarely transferred to an intensive care unit (ICU) [13], both also found in our study. We speculate whether advanced age to some extent reduces the chances of patients receiving proper clinical monitoring and timely antibiotic treatment. In contrast, in our results there was no association between age and the degree of missing data for bilirubin, arterial lactate and international standardized ratio (results not shown), indicating that the adequacy of monitoring was the same, irrespective of age.

Despite the efforts to broaden the understanding of sepsis diagnosis beyond SIRS, this entity is still used as rule in criteria for transfer to ICU and for aggressive treatment. Elderly patients’ subtle presentation of infection makes the sensitivity of SIRS a matter of concern. Studies of the prognostic value of SIRS in sepsis are scarce due to the fact that SIRS itself is generally part of the inclusion criteria. One study of ICU-patients with bacteremia caused by *Pseudomonas aeruginosa* and *Enterococcus* found no differences in SIRS between elderly and younger patients [26]. In our material, the sensitivity for organ failure of three SIRS criteria was lower than that of two criteria in the elderly, whereas the confidence intervals overlapped in the younger patients. If absence of SIRS is used as an exclusion criterion for tight observation and aggressive treatment, a prognostic sensitivity of about 60% is hardly satisfactory, and this finding is clearly clinically relevant.

The usefulness of CRP in sepsis diagnosis has been questioned [27]. A recent meta-analysis on a mixed group of ICU patients found that early CRP did not predict outcome, whereas CRP at Day 2 following admission did [28]. Another study recently found that CRP is a useful marker of sepsis resolution [29]. In our study, CRP at cut-off value 80 m mg/L was not associated to in-hospital mortality within 14 days after admission, whereas CRP at cut-off value 200 mg/L was, but only in the univariate analysis. Interestingly, we found that the sensitivity of a high CRP-level in the diagnosis of organ failure in bacteraemia is lower in elderly patients than in younger patients.

Age was clearly associated with both early organ failure and in-hospital mortality, reflecting the findings of other studies [3,5,6]. It is possible that these findings could have been confounded by age-associated clinical presentation hampering the diagnostic work-up and the timeliness of treatment, rather than age being considered a risk factor in itself. Early diagnosis of sepsis is a prerequisite for early goal directed therapy, which improves outcome [30]. Decline in functional status, together with fever, defined by lower cut-off values than those used in SIRS and SAPS [15,16], are important criteria for suspecting infection in older patients [31]. Decline in functional status includes new or increasing confusion, incontinence, falling, deteriorating mobility, reduced food intake, or failure to cooperate with staff, which partly corresponds to “atypical symptoms” assessed in our study. It might constitute a problem that such “soft variables” are not included in mortality-prediction rules for elderly ED patients with infection [32]. Our study indicates that such clinical presentations may be associated with severity of infection, though not statistically significant in the multivariate full model.

A subtle presentation may complicate the diagnosis of infections in elderly patients [33]. In our material, clinical judgment on general health in the ED independently predicted organ failure. The International Sepsis Definition Conference in 2001 acknowledged the value of clinical judgment: “*Few*, *if any*, *patients in the early stages of the inflammatory response to infection are diagnosed with sepsis via four arbitrary criteria*. *Instead the clinician goes to the bedside*, *identifies a myriad of symptoms and regardless of an evident infection declares the patient to* “*look septic*” [21]. The updated Surviving Sepsis Campaign guidelines acknowledge clinical judgment even stronger: “*Recommendations from these guidelines cannot replace the clinician*’*s decision*-*making capability when he or she is provided with at patients*’ *unique set of clinical variables*”. However, studies on the effectiveness of clinical judgment in predicting prognosis are scarce. Several studies on severe infection and sepsis did not include “soft” variables, and instead focused on biomarkers and score systems.

Traditionally, prognostication in critical illness has relied heavily upon measures of acute physiological derangements upon admission to ICU, as scoring systems do not integrate pre-hospital functional status, severity of comorbid illness, disability or frailty [34]. Cancer, diabetes or cardiovascular disease are the most important factors for health-related quality of life after critical illness [35]. Comorbidity, quantified by the Charlson comorbidity index, is a prognostic factor for in-hospital mortality [36]. In our study, the number of comorbid illnesses and comorbidity dichotomized at ≥ 1 illness were independently associated to early organ failure and in-hospital mortality, respectively.

The main strength of the study was that patients were recruited non-selectively and from a mixed group of hospital patients. All patients who were admitted to the hospital over more than a decade were included in the study population. A major weakness is that data were retrospectively collected. Thus, systematic information on adequacy of antimicrobial treatment was missing and was therefore omitted from the analyses. Furthermore, the validity of the estimated number of failing organs may be uncertain. We may have overestimated organ failure because we did not exclude failure in the organ that was considered primary source for infection. Conversely, organ failure may also have been underestimated because data on liver function and hematological markers as well as markers of peripheral perfusion were unsystematically registered and therefore excluded. The survival curve by number of failing organs (the middle part of Figure 2), however, is very similar to 1-year survival curves found by others [37]. We identified leukopenia as a risk factor for poor prognosis in the multivariate models, which corresponds well with neutropenia being one of the clinical risk factors for mortality in sepsis found in several trials [38]. We believe these findings support the importance of the “geriatric-focused results” found in our study.

## Conclusions

Elderly patients with bacteremia more often present with atypical symptoms and reduced general condition. SIRS have poorer sensitivity for identifying severe infection in these patients, and should be less emphasized when assessing the risk of sepsis in elderly patients. Advanced age and comorbidity are risk factors for both early organ failure and in-hospital mortality. An uncertain clinical presentation, however, does not seem associated with in-hospital mortality. Irrespective of age, simple observations such as the subjective judgment of decline in general health, as well as single aspects of SIRS such as tachypnea, hyperventilation and leukopenia, alongside with indicators of organ failure, are crucial when evaluating patients with possible severe infection. Because the clinical presentation is often atypical in advanced age, these clinical evaluations may be seen as keys to safer care for elderly patients with severe infection.

### Key messages

● high age and comorbidity are risk factors for poor outcome in severe infection.

● reduced general health at admittance is underestimated as a prognostic tool.

## Abbreviations

OR: Odds Ratio; CI: Confidence Interval; ED: Emergency Department; SIRS: Systemic Inflammatory Response Syndrome; SIRS-2: ≥ 2 SIRS-criteria; SIRS-3: ≥ 3 SIRS-criteria; SAPS: Simplified Acute Physiology Score; CRP: C-reactive Protein; SOFA: Sequential Organ Failure Assessment; RIFLE: Risk, Injury, Failure, Loss, End-stage kidney; ICU: Intensive Care Unit.

## Competing interests

The authors declare that they have no competing interests.

## Authors’ contributions

ALW participated in the concept and design of the study, gathered data on bacteraemia, serum markers of infection, clinical data on presentation of infection, performed the analyses and participated substantially in the writing of the manuscript. OD participated in concept and design, interpretation of the data and writing of the manuscript. KKM participated in design and writing of the manuscript. URD assisted in the data interpretation and participated substantially in the writing of the manuscript. TBW participated in design, data analysis and substantially in the writing of the manuscript. All authors read and approved the final manuscript.

## Pre-publication history

The pre-publication history for this paper can be accessed here:

http://www.biomedcentral.com/1471-2334/13/346/prepub

## Supplementary Material

Additional file 1 Criteria for acute organ failure. Click here for file
