# Supplementary material for: Age-related differences in symptoms, diagnosis and prognosis of bacteremia
Source: BMC Infect Dis. 2013 Jul 24;13:346. doi: 10.1186/1471-2334-13-346 (PMC3733624; doi:10.1186/1471-2334-13-346)
Supplement: Additional file 1 — Criteria for acute organ failure. [file 1471-2334-13-346-S1.docx]

| **Additional file 1 Criteria for acute organ failure** | |
| --- | --- |
| **Acute circulatory failure** | Systolic blood pressure below 90 mmHg in spite of >1.5L intravenous liquid transfusion or/and treatment with vasopressive medication |
| **Acute respiratory failure** | Need of respiratory assistance and/or arterial pressure of oxygen divided by the fraction of oxygen <40kPa |
| **Acute renal failure** | Single creatinine value ≥ 300 µmol/L with no history of renal failure (corresponding to SOFA score 3 or worse); or if a history of moderate renal failure was known, an increase in creatinine level > 50 µmol/L from prior value or > 50 µmol/L decrease during the first day of admittance; Patients with a known history of severe chronic renal failure were not considered to experience acute worsening |
| **Acute coagulatory dysfunction** | Platelet number below 100 x 10^3^/mm^3^ |
| **Impaired consciousness** | Signs of agitation, somnolence, stupor. Coma. |
